# Functional Antagonism of Sphingosine-1-Phosphate Receptor 1 Prevents Harmaline-Induced Ultrastructural Alterations and Caspase-3 Mediated Apoptosis

**DOI:** 10.21315/mjms2019.26.4.4

**Published:** 2019-08-29

**Authors:** Narjes Dahmardeh, Mohammad Shabani, Mohsen Basiri, Taj Pari Kalantaripour, Majid Asadi-Shekaari

**Affiliations:** 1 Department of Anatomical Sciences, Afzalipour Medical Faculty, Kerman University of Medical Sciences, Kerman, Iran; 2 Department of Anatomical Sciences, Faculty of Medicine, Zabol University of Medical Sciences, Zabol, Iran; 3 Neuroscience Research Center, Neuropharmacology Institute, Kerman University of Medical Sciences, Kerman, Iran; 4 Department of Physiology, School of Medicine, Kerman Branch, Islamic Azad University, Kerman, Iran

**Keywords:** harmaline, essential tremor, fingolimod, apoptosis, rats

## Abstract

**Background:**

There is a meaningful necessity for a targeted therapy of essential tremor (ET), as medications have not been developed specifically for ET. For nearly a century, many drugs have been applied in the treatment of tremor but the drug treatment of ET remains still unknown. Some potential therapeutic factors such fingolimod (FTY720) can be effectively used to treat ET in animals. In the present research, the effect of FTY720, the immunomodulatory sphingosine 1-phosphate (S1P) analog, on degeneration of cerebellar and olivary neurons induced by harmaline in male rats was investigated.

**Methods:**

The animals were allotted into control dimethyl sulfoxide (DMSO), saline + harmaline [30 mg/kg, intraperitoneally, (i.p.)], harmaline + FTY720 (1 mg/kg, i.p, 1 h and 24 h before harmaline injection) groups (*n* = 10). The cerebellum and inferior olive nucleus (ION) were studied for neuronal degeneration using immunohistochemistry (IHC) and ultrastructural study by transmission electron microscopy (TEM) techniques.

**Results:**

Harmaline caused neuronal cell loss, caspase-3 mediated apoptosis, astrocytosis and ultrastructural changes in cerebellar Purkinje cells and inferior olive neurons. FTY720 exhibited neuroprotective effects on cerebellar Purkinje cells and inferior olivary neurons.

**Conclusion:**

These results suggest that FTY720 has potential efficacy for prevention of ET neurodegeneration and astrocytosis induced by harmaline in male rats.

## Introduction

Essential tremor (ET) is one of the most prevalent neurological disorder in adults (1, 2), characterised by kinetic and postural oscillation involving a body part or more (3). Its prevalence estimates ranging from 0.4% to 5% (4). Despite this high prevalence, the pathogenesis and etiology of ET are not completely known (5). The harmaline as an indole alkaloid, is a plant-derived metabolite used to induce tremor in animals (6). Harmaline administration induces a high frequency tremor and produces abnormal motor behaviour in rats (7).

Harmaline, as apsychoactive alkaloid, has excitatory effects on the central nervous system (CNS) with increased firing rate in the inferior olivary nucleus neurons (ION) (8). The ION may play the primary role in the producing tremor in ET (9). It was assumed that harmaline with an increase of neuronal firing in the inferior olive, leading to release glutamate from climbing fibers that synapses with Purkinje cells. The repetitive of an excitatory neurotransmitter release, produces excitotoxic damage and degeneration in Purkinje cells (8).

Recent controlled post-mortem evidence documented that ET is related to the histological changes of the neural cells in the cerebellum (4, 10). These changes were detected in Purkinje cells including swellings of axons (11), heterotopic displacement (12) and cell death (13). Considering the absence of understanding the basic mechanism of tremors, it will be challenging to develop pharmacological agents with anti-tremor activity (8).

Fingolimod (FTY720) is an innovative oral drug approved in 2010 for therapy of MS patients (14). The sphingosine kinase phosphorylates FTY720 into an activated form, FTY720-P (15) and regulates several cellular responses (16). Emerging evidence indicates extending the success of FTY720 in the CNS beyond immunomodulation to include other multiple sclerosis (MS) pathophysiology aspects, such as an influence on the blood-brain barrier (BBB) and glial repair mechanisms that could eventually play role in the restoration of nerve function (17).

Tremor induced by harmaline is one of the animal models of transient action tremor (18, 19). In this model, inferior olive activation transfers to the cerebellar Purkinje cells through climbing fibers. Studies which evaluated the pharmacological profile (20, 21) influenced brain areas (22, 23) and the phenomenological features of harmaline-induced tremor (21, 24) indicated the usefulness of this model in assessing the clinical efficacy of putative therapies in ET.

FTY720 has shown anti-inflammatory and neuroprotective roles in different animal models of the CNS disorders, in addition to its immunomodulatory functions in MS (25). In the behavioural study, Dahmardeh et al., showed that FTY720 ameliorated motor impairments of ET induced by harmaline in rats (26). This study aimed to investigate the effect of FTY720, the immunomodulatory sphingosine 1-phosphate (S1P) analog, on the harmaline-induced destruction of cerebellar and olivary neurons in male rats.

## Methods

### Animals

Forty male Wistar rats weighing 40 g–60 g were under a 12 h light-dark cycle with ad libitum access to food and water. The animals were allotted into control dimethyl sulfoxide (DMSO), saline + harmaline [30 mg/kg, intraperitoneally, (i.p.)], harmaline + FTY720 (1 mg/kg, i.p., 1 h and 24 h before harmaline injection) groups (*n* = 10). All efforts were performed to minimise animal suffering.

### Preparation and Administration of Drugs

Harmaline hydrochloride dihydrate (Sigma, Germany, 30 mg/kg) and FTY720 (Sigma, Germany, 1 mg/kg; i.p.) were dissolved in normal saline and DMSO (1% v/v) on the day of the experiment, respectively. The FTY720 administered 1 h (FTY720/1h) and 24 h (FTY720/24h) before harmaline injection. Vehicles as pre-administration, injected to the harmaline group 1 h before harmaline injection, to keep the same number of injections in all groups. Maximum volume for intraperitoneally injection of drugs was 1 mL.

### Histological Analysis

The rats were euthanised under deep anesthesia and cerebellar cortex and the ION tissues were fixed in 10% buffered formalin for 24 h and then were processed to prepare 5 μm thick paraffin sections for hematoxylin-eosin staining and immunohistochemistry (IHC) study.

### Hematoxylin-Eosin Staining Method

The tissues were hydrated and stained with Harris hematoxylin and eosin Y. Completely dehydrated sections were cleared with xylene and mounted with entellan (27).

### IHC Study

For IHC, neurons were controlled in the cerebellar cortex and the ION by primary antibodies: GFAP and caspase-3. The sections were dipped with Tris-buffer saline (TBS), dehydrated in ethanol, hydrated in distilled water. To the blocking non-specific binding sites, using a solution of 0.3% Triton and 5% goat serum dissolved in TBS for 3 h. Then samples were immersed in methanol 3% hydrogen peroxide solution for 1 h at room and endogenous peroxidase was inactivated. The sections were incubated using the primary antibodies, including anti-GFAP (1:500; Sigma, St. Louis, MO) and anti-caspase-3 (1:300; Santa Cruz, CA, USA) antibodies (1:300) at 4 ºC overnight. The samples were washed in TBS three times. Subsequently, these samples incubated with the secondary antibodies (mouse/rabbit) conjugated with peroxidase for 1 h at ambient temperature. Sections were washed in water, immersed in copper sulphate (3,3′-diaminobenzidine) DAB enhancer (4 min), and counterstaining of the nuclei was performed with haematoxylin, dehydrated, cleared and mounted. Images were captured with a microscopic digital camera (50i) (Nikon-Japan). Cells that showed GFAP and caspase-3 immunoreactivity were manually counted in four microscopic fields (0.1070 mm^2^; 89.82 × 120.70 μm) of the IHC stained sections from cerebellum and ION. Results were presented as the average number of cells/0.10 mm^2^ (28).

### Electron Microscope Study

For transmission electron microscopy (TEM) assessment, specimens (cerebellar cortex and the ION tissues) were fixed in 2.5% phosphate-buffered glutaraldehyde (pH 7.4) and then were post-fixed in 1% osmium tetroxide in the same buffer at 4 °C, dehydrated and embedded in resin. Ultrathin sections were stained with uranyl acetate and lead citrate and photographed using TEM (Zeiss 10 EM) in the department of an electron microscope of the Kerman Neuroscience Research Center (KNRC). Semithin sections (400 nm thick) were stained with 1% toluidine blue for finding the area of interest.

### Statistical analysis

One-way analysis of variance (ANOVA) was used to analyse the data with Tukey’s post-hoc, as a pairwise comparison between groups. All data were expressed as the mean ± SEM and *P* < 0.05 was considered statistically significant.

## Results

**Figure 1:**
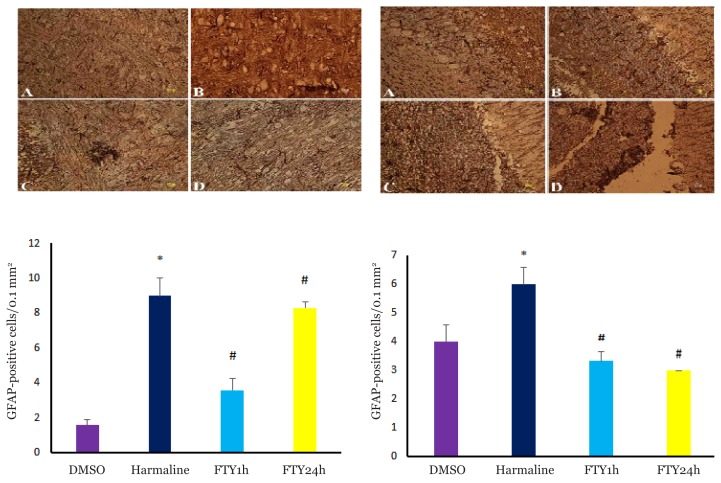
Immunohistochemical analysis of GFAP^+^ cells in the cerebellum of rats (right panel) and ION (left panel). DMSO (A), harmaline (B), FTY720/1h (C) and FTY720/24 h (D). Scale bar: 10 μm. Bottom panel, the bar graph shows the quantitative analysis of GFAP+ cells in the cerebellum and ION of rats in different groups. Data are presented as means S.E.M. In the cerebellum * was considered for *P* < 0.045 compared with DMSO group and # was for *P* < 0.006 compared with harmaline group. In ION * was considered for *P* < 0.001 compared with the DMSO group and # was considered for *P* < 0.001 compared with harmaline (one-way ANOVA with Tukey’s post-hoc test for all comparisons).

The results indicated that the number of GFAP^+^ astrocytes was significantly increased in harmaline group compared to DMSO group in the cerebellum (*P* < 0.045) (Figure 1, right panel) and ION (*P* < 0.001) (Figure 1, left panel), and the number of these cells was significantly lower in FTY720 group than harmaline group in the cerebellum (*P* < 0.006) and ION (*P* < 0.001).

**Figure 2:**
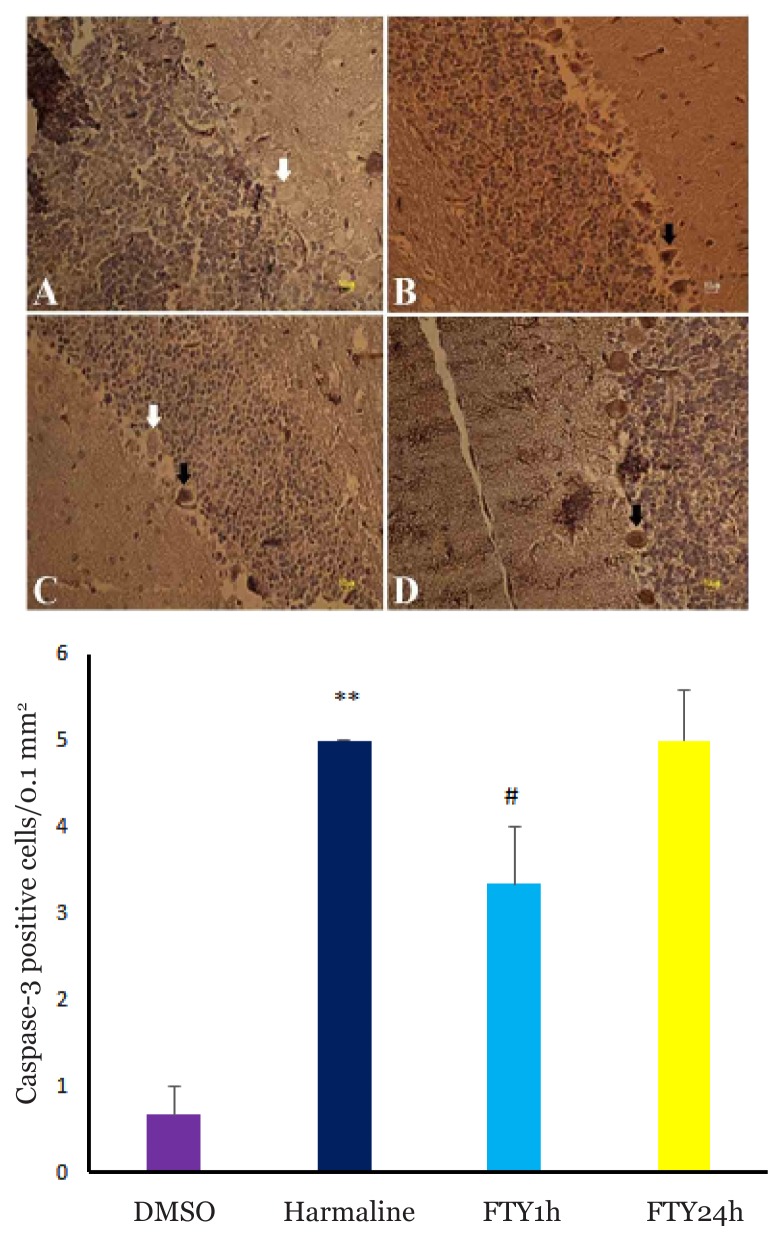
Upper panel, immunohistochemical analysis of caspase-3^+^ cells in the cerebellum. DMSO (A), harmaline (B), FTY720/1h (C) and FTY720/24 h (D). Scale bar: 10 μm. White arrow shows normal cell (neuron) and black arrow shows caspase-3+ neuron. Bottom panel, the bar graph shows the number of caspase-3^+^ neurons was increased in harmaline group and pretreatment with FTY attenuated caspase-3^+^ cells. ** *P* < 0.001 compared with DMSO group. # *P* < 0.031 compared with harmaline group (one-way ANOVA with Tukey’s post-hoc test for all comparisons).

**Figure 3:**
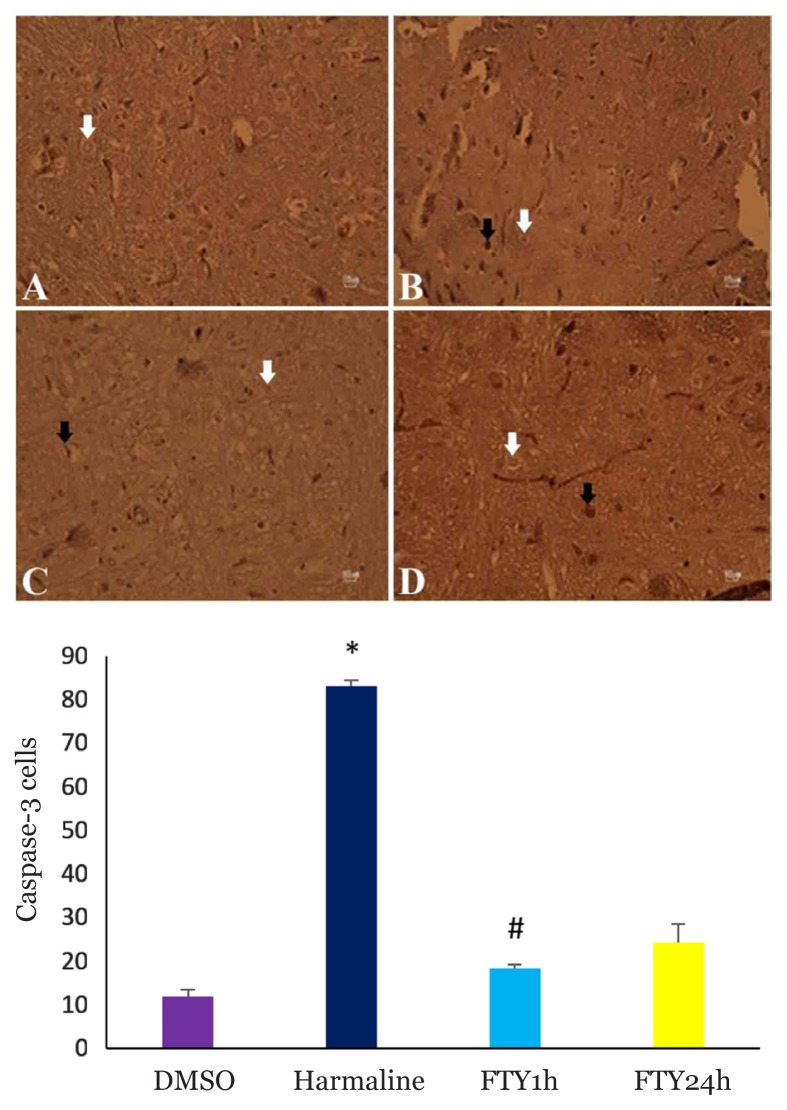
Upper panel, immunohistochemical analysis of caspase-3^+^ in inferior olive of the rat. DMSO (A), harmaline (B), FTY720/1h (C) and FTY720/24h (D). Scale bar: 10 μm. White arrow shows normal cell (neuron) and black arrow shows apoptotic neuron. Bottom panel, bar graph show the number of caspase-3^+^ neuron was increased in harmaline group and pretreatment with FTY attenuated caspase-3+ cells. * *P* < 0.001 compared with DMSO group. # *P* < 0.008 compared with harmaline group (one-way ANOVA with Tukey’s post-hoc test for all comparisons).

Harmaline administration resulted in increasing the number of caspase-3^+^ Purkinje cells (*P* < 0.001) (Figure 2) in cerebellum and pretreatment with FTY720 significantly reduced the number of caspase-3^+^ cells (*P* < 0.031). In addition, the number of caspase-3^+^ neurons in ION was increased in harmaline group (*P* < 0.001) (Figure 3) and FTY720 pre-treatment attenuated the number of these cells (*P* < 0.008).

**Figure 4:**
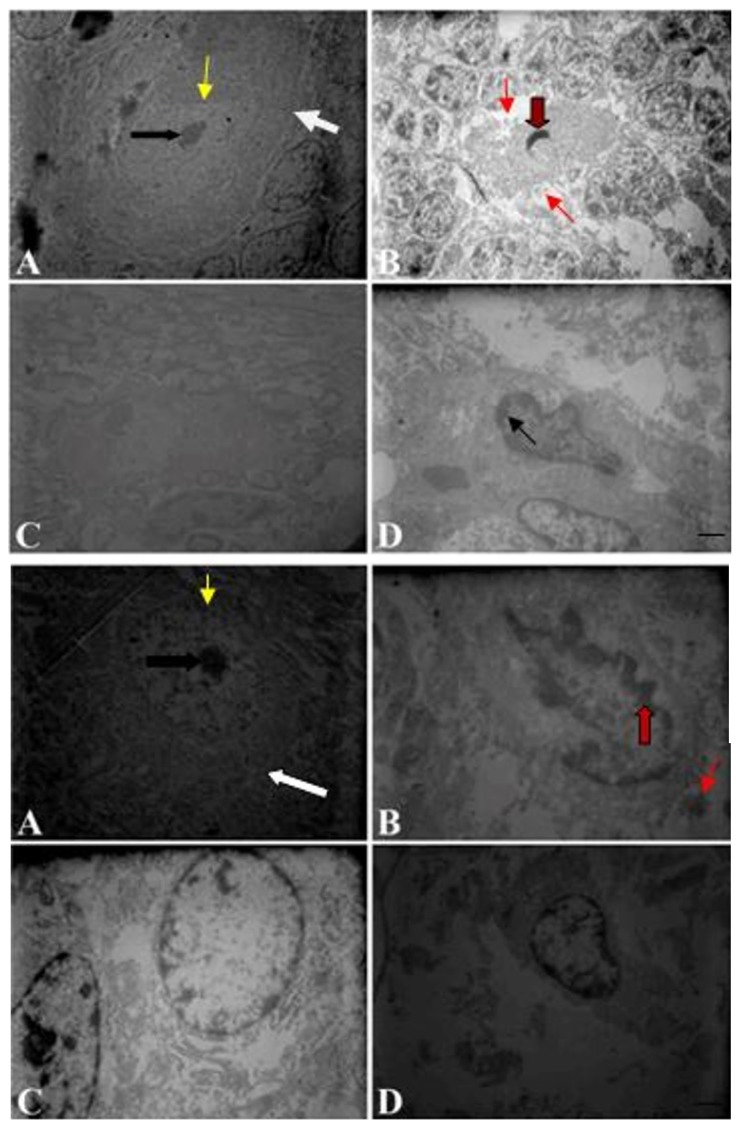
Upper panel, an electron micrograph of the ultrastructure of Purkinje cells in the cerebellum of rats. Bottom panel, an electron micrograph of the ultrastructure of inferior olive neurons of rats. The prominent nucleolus (black arrow), intact nucleolemma and cell membrane (yellow and white arrows) were visible in part A. Note ultrastructural alterations including chromatin aggregation (dark red arrow) nuclear deformity and apoptotic bodies (narrow red arrow) in part B. Control (DMSO) (A), harmaline (B), FTY720/1h (C) and FTY720/24h (D). Scale bar: 1.5 μm.

In DMSO treated rats, Purkinje cells of the cerebellum and ION neurons had normal morphology including intact cell membrane, clear nucleus, intact nuclear membrane, dispersed chromatin and prominent nucleolus. Harmaline induced eminent ultrastructural changes in Purkinje cells of the cerebellum and ION neurons. Cell and nuclear shrinkage, chromatin margination, apoptotic bodies and dark cytoplasm formation were the most ultrastructural findings in neurons of the harmaline treated group. In FTY720/1h group, the ultrastructure of most neurons was preserved. In this group, apoptotic bodies were not observed. On the other hand, chromatin aggregation, dark cytoplasm and shrinkage of the nuclei were found in FTY720/24h group (Figure 4).

## Discussion

In most patients suffering from ET, the illness is not recognised and never cured. ET is a prelavent movement complaint. The severity of the tremor and handicap differ extensively. Moreover, because of side-effects or poor efficacy, many patients don’t follow the treatment (29). This study presented the innovative findings regarding the tremor harmaline model, and the impacts of FTY720 on this disorder.

According to our data, harmaline increased caspase-3 positive cells in cerebellum and ION and the neuroprotective effects of FTY720 was in agreement with other studies (30–32). Furthermore, electron microscopy study indicated that classical morphological features of apoptosis in neurons of cerebellum and ION including chromatin condensation and apoptotic bodies. FTY720 administration led to neuronal morphology preservation in the treated group. In addition, administration of FTY720 decreased caspase-3 positive cells in the cerebellum and ION neurons. In other works, Rolland et al., demonstrated that FTY720 in model of cerebral ischemia has anti-apoptotic effects (16). Cipriani et al., also reported that FTY720 has neuroprotective properties against NMDA (N-methyl-d-aspartic acid)-mediated excitotoxicity (33).

In vitro studies demonstrate that aspartate-specific cysteine proteases (caspases) are effectors of apoptosis (34). In neurons, several lines of evidence show that caspase-3, a 32 kDa cytosolic protein, plays a key role in the executive phase of apoptosis (35). Neuronal death in experimental models of several acute and chronic neurodegenerative disorders was related to activation of caspase-3 (36).

NMDA receptors (NMDAR) constitute a major group of glutamate-gated ion channels and contribute to neurodegeneration related to excitotoxicity significantly (37). Since it has been indicated that ION and cerebellum involve a high density of (NMDAR) (38), it may be emphasised that functions abnormally of NMDAR in ET (39) can be treated by NMDA antagonists (19). The excitotoxic cellular death in the brain is considered as a series of necrotic, apoptotic, and autophagic morphologies (40).

Recent studies indicated that harmaline in ION increases T-type calcium pulses. Therefore, T-type calcium channels blockers may have therapeutic results in tremor disorders (41). Dahmardeh et al. showed that FTY720 reduced the intensity of tremor and locomotor disorders (26) which may show its anti-tremorogenic efficacy. Via chemical ablation in olivo-cerebellar fiber may be prevent the degeneration of Purkinje cells. It has been highlighted that the excitotoxic destruction of the Purkinje cells may be caused by an excitatory amino acid release from the climbing fiber (42). Harmaline can increase synaptic activity of climbing fibers creating from ION (43). Excitatory amino acids levels in the cerebellum increase through the climbing fiber system activation. It is, therefore, possible that these acids are responsible for harmaline tremor mechanism (44).

Astrocytes are responsible for maintaining the brain homeostasis. Their processes through expressing the numerous receptors for neurotransmitters, some transporters, cytokines, and growth factors, modify the neuronal activity (45). Given that the variability of basic functions applied by astrocytes to support neurons, it has been made clear that astrocyte impairment play a key role in neuronal dysfunction in several neurodegenerative illnesses, including amyotrophic lateral sclerosis, Alzheimer’s and Huntington diseases (46). In the pathological conditions, the response of astrocytes is very heterogeneous. It was recently shown that glio-transmission regulates the trafficking and surface expression of the NMDAR (47).

For many key CNS functions, NMDAR is essential (48). It has been shown that NMDAR activity in astrocytes exerts neuronal antioxidant protection (49).

In consistent with previous studies, our study results revealed that harmaline increased astrocyte number and FTY720 diminished astrocytes number in treated groups. Harmaline may by acting on the NMDAR, induce tremor and causing cell death (50). Deogracias et al., reported that FTY720 can reduce cytotoxicity induced by NMDA in a brain-derived neurotrophic factor dependent method (51). So it can be predicted that FTY720 might reduce the effects of harmaline by blocking NMDAR (52). Besides, Hoffmann et al., suggested that neuroprotective effects of FTY720 on astrocytes may be through the induction of neurotrophic factors and inhibition of inflammatory genes expression and FTY720 effects might be mediated by astrocytes (53). Furthermore, Van Doorn et al. showed that FTY720 inhibit production of inflammatory cytokines in human astrocytes (54). It is known that under pathological conditions, astrocytes release pro-inflammatory cytokines and FTY720 treatment can reduce cerebral cytokine levels (55). FTY720 may also act with restricting the release of neurotoxic mediators from astrocytes, in addition to the therapeutic effects through reducing the inflammatory cell influx into CNS (55).

## Conclusion

The most prominent results of the present work are as the following: harmaline can induce neurodegeneration in Purkinje cells of the cerebellum and ION and FTY720 attenuate harmaline induced neuronal injury, probably as results of the anti-apoptotic and anti-inflammatory effects. To sum up, the FTY720 seems to be an encouraging therapeutic factor against harmaline induced neurodegeneration changes in rat cerebellum and ION.
